# Risk of prostate cancer in a population-based cohort of men with coeliac disease

**DOI:** 10.1038/bjc.2011.536

**Published:** 2011-12-01

**Authors:** J F Ludvigsson, K Fall, S Montgomery

**Affiliations:** 1 Department of Paediatrics, Örebro University Hospital, Örebro 701 85, Sweden; 2 Clinical Epidemiology Unit, Department of Medicine, Karolinska Institutet, Stockholm 171 76, Sweden; 3 Clinical Epidemiology and Biostatistics Unit, Örebro University Hospital, Örebro University, Örebro 701 85, Sweden; 4 Department of Epidemiology, Harvard School of Public Health, Boston, MA 02115, US; 5 Department of Primary Care and Public Health, Charing Cross Hospital, Imperial College, London W6 8RF, UK

**Keywords:** coeliac disease, cohort study, inflammation, prostate cancer

## Abstract

**Background:**

Prostate cancer (PC) is a leading cause of fatal cancer in men in developed countries. Coeliac disease (CD) has previously been linked to a raised cancer risk, and changes in some exposures following a CD diagnosis might hypothetically raise PC risk.

**Methods:**

We identified 10 995 patients with CD who had undergone a small intestinal biopsy in 1969–2007. Statistics Sweden then identified 54 233 age-matched male reference individuals from the general population. PC data were obtained from the Swedish Cancer Register. Hazard ratios (HRs) for PC were estimated using Cox regression analysis.

**Results:**

During follow-up, 185 individuals with CD (expected *n*=200) had an incident diagnosis of PC. This corresponds to a HR of 0.92 (0.79–1.08) (with 95% confidence interval) and an absolute risk reduction of 15/100 000 person-years among those with CD. An increased risk was not observed even when identification of PC began 5 years after biopsy.

**Conclusion:**

Our conclusion is that a CD diagnosis does not represent an increased risk for PC.

Prostate cancer (PC) is one of the leading causes of cancer mortality in men in industrialised countries (Ferlay *et al*, 2010). Established risk-factors for PC include increasing age, ethnic origin, and hereditary/familial factors. Although the great variability in incidence across different parts of the world can be attributed largely to differences in diagnostic intensity, the over 10-fold variation in mortality rates between low-risk countries in Asia and high-risk countries, such as Sweden (Ferlay *et al*, 2010), suggests that environmental factors, such as diet, may account for some of the observed variation (Shimizu *et al*, 1991; Hsing and Devesa, 2001).

Coeliac disease (CD) is an immune-mediated disease that occurs in 1% of the Western population (Dube *et al*, 2005). It is characterised by small intestinal inflammation and villous atrophy (VA) (Ludvigsson and Green, 2011). The only available treatment consists of a gluten-free diet where the individual avoids wheat, barley, and rye (Kupper, 2005), whereas consumption of oats is safe for patients with CD (Janatuinen *et al*, 1995).

Earlier research has shown a relative risk for PC in CD of around 1, with two studies suggesting a lower risk of PC (England: standardised incidence ratio=0.67; 95% confidence interval (CI)=0.18–1.73) (Goldacre *et al*, 2008) (Sweden: 0.7; 95% CI=0.4–1.2) (Askling *et al*, 2002), and one study suggesting a positive association between positive endomysial antibodies (‘gluten sensitivity’) and PC (Northern Ireland: 1.78; 95% CI=0.00–4.24) (Anderson *et al*, 2007). All of these studies were, however, based on <20 cases of PC. Additionally, there are single case-reports of PC in CD (Green *et al*, 2003; Card *et al*, 2004), but with no relative risks reported.

An association between CD and PC could potentially be mediated by several factors including dietary changes following diagnosis, as patients with CD are prescribed a gluten-free diet, which includes low intake of rye, wheat, barley, and refined grain. Dietary change could potentially influence PC risk, although the link between intake of whole grains and PC is unclear. Two small intervention studies among PC patients have reported associations between increased rye intake and tumour growth in terms of reduced PSA-levels and increased apoptosis (Bylund *et al*, 2003; Landberg *et al*, 2010), while observational studies of whole grain consumption and PC risk show inconsistent results (Chatenoud *et al*, 1998; Lewis *et al*, 2009; Egeberg *et al*, 2011).

In this study, we tested the hypothesis that men with a diagnosis of CD have a higher risk of PC. This was tested using longitudinal Swedish register data for 10 995 men with CD and 54 233 age-matched men in the comparison cohort.

## Materials and methods

We used national Swedish data from biopsy reports to identify patients with CD. These data were linked to the Swedish Cancer Register in order to examine the risk of PC.

### PC

PC was defined according to the International Classification of Disease (ICD) 7-code 177 in the Swedish Cancer Register (equivalent to ICD-8 and 9: 185 and ICD-10: C61).

### CD

From October 2006 to February 2008, data from small intestinal biopsy reports were collected from each of Sweden's 28 pathology departments. The biopsies were performed between 1969 and 2008. CD was defined as having a biopsy report with VA (equivalent to Marsh grade 3; see Supplementary Appendix) (Marsh, 1992). We did not require positive serology for a CD diagnosis, but an earlier validation study found that among patients with available CD serology data at the time of biopsy, 88% had positive serology (Ludvigsson *et al*, 2009a). The validation study also found that 95% of patients with VA had CD according to medial records (*n*=108/114) and that 79% of patients had gastrointestinal symptoms at the time of biopsy.

Local IT personnel conducted the searches for relevant biopsy reports and data on the arrival dates of the biopsies, personal identity number (Ludvigsson *et al*, 2009c), morphology according to the Swedish SnoMed classification codes (for a translation into international classification systems, see Supplementary Appendix), and topography (duodenum/jejunum). These data were then delivered to the researchers. The CD cohort used here was based on the 29 096 individuals with CD and was examined for overall mortality in a recent paper by our group (Ludvigsson *et al*, 2009b). The number of age- and sex-matched controls (identified by the government agency statistics) individually matched with patients with CD in that study was 144 522.

The CD study sample was then restricted to 11 091 male CD patients (18 005 females excluded) (Ludvigsson *et al*, 2009b). We then excluded individuals with CD who had a biopsy after 31 December 2007 (*n*=5) because our follow-up ended on 31 December 2007 (cancer data available until this date), individuals with a diagnosis of PC before CD diagnosis (*n*=90). Finally, there was left one individual with CD whose matched comparators had been excluded for any of the above reasons.

Of the initial 144 522 controls, 55 300 were males (89 222 females were excluded). We also excluded controls that entered the study after 31 December 2007 (*n*=25), those with a diagnosis of PC before CD diagnosis (*n*=480), and controls whose matched index individuals with CD had been excluded at some stage (*n*=562).

The final sample consisted of 10 995 men with CD and 54 233 in the age-matched male comparison cohort.

### Statistics

Cox regression was used to estimate relative risks. In our statistical model, we used internal stratification for age at the time of the first biopsy (and corresponding age in reference individuals), calendar period, and county. Our analysis therefore took into account the influence of age, calendar period, and county (similar to the effect of use of risk-sets in conditional logistic regression). Follow-up time started on the date of the first biopsy with VA and on the corresponding date in matched reference individuals. It ended with a diagnosis of PC, death, emigration, or on 31 December 2007, whichever came first. In reference individuals, follow-up could also end if the individual underwent a small intestinal biopsy. We used log-minus-log curves to test the proportional hazards assumption (Figure in Supplementary Appendix showing that this condition was fulfilled). We also evaluated the risk of PC stratified by follow-up time (<1 year, 1 to <5 years, and ⩾5 years), sex, age at CD diagnosis (0–19, 20–39, 40–59, and ⩾60 years at first biopsy), and calendar period of the first biopsy (−1989, 1990–1999, and 2000 until present). Death due to PC (based on underlying cause of death according to death certificates) was used as an alternative outcome in a subanalysis in an attempt to identify a more aggressive PC phenotype. Incidence rates were calculated using the number of first PC events divided by the number of person-years at risk. The expected number of cases was derived from the observed number of cases divided by the hazard ratio (HR). In this way, the expected number of cases is based on the age and sex distribution of the CD cohort.

We identified individuals with type 1 diabetes using the Swedish Hospital Discharge Register (see Supplementary Appendix for ICD codes) (Ludvigsson *et al*, 2011). Type 1 diabetes is associated with CD (Bao *et al*, 1999; Smyth *et al*, 2008), but inversely associated with PC, so concomitant type 1 diabetes could therefore potentially hide a positive association between CD and PC. A subanalysis excluded all individuals with a diagnosis of type 1 diabetes, irrespective of when the diagnosis was made.

In another subanalysis, we adjusted for education using seven *a priori* educational categories determined by Statistics Sweden (<9 years, 9–10 years, ⩽2 years of high school, ⩾3 years of high school, <3 years of college/university, ⩾3 years of college, and postgraduate studies). In a third subanalysis, we adjusted for country of birth (Nordic and non-Nordic countries).

Statistical significance defined as 95% CI for risk estimates not including 1.0. SPSS 18.0 software (SPSS Inc., Chicago, IL, USA) was used for the statistical analysis.

## Results

### Background data

The majority of the participants with CD had a biopsy from 1990 onwards and entered the study at this time, as did the matched comparison cohort. The median age at diagnosis of PC was 71 years in men with CD and 72 years in the comparison cohort. Other characteristics of the study participants are listed in Table 1. Some 3.8% of the individuals with CD but only 0.4% of the matched controls had a diagnosis of type 1 diabetes before the end of follow-up (*P*<0.001, *χ*^2^-test).

### CD and subsequent PC

During follow-up, there were 185 diagnoses of PC (expected *n*=200), corresponding to a HR of 0.92 (95% CI=0.79–1.08) (Table 2). The risk estimate did not change notably when we excluded individuals with a diagnosis of type 1 diabetes (0.93; 0.79–1.09). Nor did it change notably when we adjusted for country of birth (0.92; 0.78–1.07) or education (0.93; 0.78–1.12).

When we excluded the first year of follow-up to minimise the risk of surveillance bias, the HR was little changed (0.89; 0.75–1.06). After 5 years of follow-up, giving sufficient time for an influence of the diet, the HR for PC in CD was 1.00 (Table 2).

The differences in PC risk by age at CD diagnosis were not statistically significant (*P*-value for interaction: 0.197) (Table 3). There were no notable differences in risk estimates for PC by calendar period (*P*-value for interaction: 0.926).

When death due to PC, indicating more aggressive disease, was used as an alternative outcome (with 21 deaths among CD patients and an expected of 36), the HR for the association of CD with PC-related death remained below 1.00 (data not shown).

## Discussion

This study found no association between CD and PC using longitudinal data. It is one of the first large-scale studies of this subject and it used histology from intestinal biopsies to identify CD reliably. Our findings are consistent with the two general population-based studies from England (Goldacre *et al*, 2008) and Sweden (Askling *et al*, 2002), although this study had substantially more statistical power to detect an association.

Although earlier studies on CD and PC have been based on as few as 2–14 positive events (Askling *et al*, 2002; Anderson *et al*, 2007; Goldacre *et al*, 2008), we observed 185 PC diagnoses in patients with CD during follow-up. The greater statistical power of our study allowed for stratification and greater precision.

CD patients in our cohort were ascertained through biopsy reports, so they are more likely to have typical CD characteristics than those identified through inpatient registers as this may signal comorbidity (Askling *et al*, 2002; Goldacre *et al*, 2008), because CD investigation does not require hospital care. Most adult gastroenterologists (96%) and all paediatricians obtain biopsies from the majority of patients with suspected CD (over 90%) to make the diagnosis. Therefore, diagnosis of CD among our study population is highly reliable. Small intestinal biopsies with VA have high specificity for CD. When two independent researchers examined more than 1500 biopsy reports with VA or inflammation, <0.3% of patients suffered from inflammatory bowel disease, which was the most common comorbidity (other than CD) (Ludvigsson *et al*, 2009a). Patients with CD are at increased risk of comorbid type 1 diabetes. We performed an analysis where all the individuals with a diagnosis of type 1 diabetes before the end of follow-up were excluded. This exclusion did not affect the risk estimates for PC.

One weakness of this study is the lack of individual data on dietary components or compliance to a gluten-free diet. Some aspects of dietary compliance could theoretically raise the risk of PC, although mucosal healing and less chronic inflammation might reduce the risk. In a validation study of 121 randomly selected individuals with VA, there were indications of low dietary compliance in 15/86 individuals (17, 95% CI=9–25%), thus with dietary compliance in 83% (Ludvigsson *et al*, 2009a). This study found no association between CD and PC (HR=0.92).

As PC may have a prolonged natural history (Schmid *et al*, 1993), we also examined risk of PC according to time since diagnosis of CD. Although there was no association between CD and PC in the first year beyond biopsy, or more than 5 years after biopsy, there was a statistically significant reduced risk of PC 1–5 years after CD diagnosis. We cannot rule out that there is a true decreased risk of PC in CD due to factors, such as lower body mass index (BMI) in patients with CD (Discacciati *et al*, 2011). In the first year after diagnosis, this may be masked by an increased ascertainment rate of PC as comorbid conditions are more likely to be detected during diagnosis or treatment. With time, the lower PC risk may not persist as mucosal healing results in increased BMI. It should be noted that the apparent reduced risk 1–5 years after CD diagnosis could represent a chance finding.

Although the total follow-up time for patients with CD in this study was 101 000 person-years, we did not have enough follow-up to estimate the risk of PC in CD diagnosed in childhood. It has been suggested that an early diagnosis of CD in childhood may protect against certain cancers (Elfstrom *et al*, 2011), but as participants in this study tended to receive a later diagnosis, this putative protection against cancer could not have concealed a positive association. Other immune-mediated diseases, such as type 1 diabetes (Kasper *et al*, 2009; Shu *et al*, 2010), ulcerative colitis (Goldacre *et al*, 2008), Crohn's disease (Goldacre *et al*, 2008), and Wegener's granulomatosis have been inversely associated with PC (Knight *et al*, 2002). This study found no association between CD and PC.

Also, adolescence represents a critical window of prostate development, where diet (Andersson *et al*, 1995) and hormonal exposure (Barba *et al*, 2008) could be important. Although we could investigate participants with a CD diagnosis at younger ages, it is important to emphasise that there was no evidence of increased PC risk among men diagnosed with CD from age 40 years: this study would have been able to detect such a risk. This indicates that middle-aged men who receive a diagnosis of CD need not be concerned that the diagnosis, or dietary changes typically associated with it, could increase their risk of PC.

Another potential weakness is our lack of data on BMI. High BMI has been inversely associated with CD (Olen *et al*, 2009), but positively associated with PC (Moller *et al*, 1994), although most prospective studies do not support this association (Rodriguez *et al*, 2007; Wright *et al*, 2007). Neither did we have any data on smoking. Smoking may increase the risk of PC (Plaskon *et al*, 2003). Although several studies have indicated an inverse relationship between smoking and CD (Vazquez *et al*, 2001; Austin *et al*, 2002), a recent study by our group found a nonsignificantly increased risk for later CD in smokers (adjusted Odds ratio=1.25; 95% CI=0.94–1.67) (Ludvigsson *et al*, 2005). Although unlikely, we cannot rule out the possibility that lower BMI and a lack of smoking among individuals with CD may have hidden a modest association between CD and PC.

PSA-testing has, since it was introduced during the 90s, resulted in a rising overdiagnosis of PC (i.e., detection of nonlethal pseudo-tumours) and it is becoming increasingly important to separate clinically significant PC from indolent disease in aetiological studies. The Swedish Cancer Registry covers essentially all incident PCs, but lacks information on tumour stage and Gleason grade. Although PSA-screening is less common in Sweden than in the United States and many other Western countries, around 40% of the cases were diagnosed with screening-detected PC in Sweden during the latter part of the study period (National Prostate Cancer Register, http://www.roc.se/prostata.asp). We tackled this through a subanalysis that used death due to PC as the outcome to identify more aggressive disease. Again, there was no raised risk associated with a CD diagnosis.

In conclusion, patients with CD seem to be at no increased risk of PC.

### Ethical approval

This project (2006/633–31/4) was approved by the Research Ethics Committee of the Karolinska Institute, Sweden on 14 June 2006.

## Figures and Tables

**Table 1:** Characteristics of the study participants

	**Matched controls**	**Patients with CD**
Total	54 233	10 995
		
*Age and follow-up*		
Age at study entry, years (median, range)	34; 0–95	35; 0–95
Age 0–19 (%)	21 734 (40.1)	4356 (39.6)
Age 20–39 (%)	8216 (15.1)	1659 (15.1)
Age 40–59 (%)	12 946 (23.9)	2605 (23.7)
Age ⩾60 (%)	11 337 (20.9)	2375 (21.6)
Entry year (median, range)	1998; 1969–2007	1998; 1969–2007
Follow-up, years (median, range)^a^	8; 0–39	8; 0–37
Follow-up, years (mean±SD)^a^	9.5±6.4	9.3±6.4
		
*Calendar year*		
-1989 (%)	8101 (14.9)	1643 (14.9)
1990–1999 (%)	22 824 (42.1)	4627 (42.1)
2000- (%)	23 308 (43.0)	4725 (43.0)
		
*Covariates*		
Nordic country of birth (%)^b^	51 241 (94.5)	10 662 (97.0)
Type 1 diabetes (%)	208 (0.4)	418 (3.8)

Abbreviation: CD=coeliac disease.

a Follow-up time until diagnosis of prostate, death from other cause, emigration, or 31 December 2007. In reference individuals, follow-up could end if the patients underwent a small intestinal biopsy.

b Sweden, Denmark. Finland, Norway, and Iceland.

**Table 2:** Risk of prostate cancer by follow-up time

**Follow-up**	**Observed events**	**Expected events**	**HR**	**95% CI**	***P*-value**	**Absolute risk/100 000 PYAR**	**Excess risk/100 000 PYAR**	**Attributable percentage**
All	185	200	0.92	0.79–1.08	0.336	182	−15	−8
Year <1	22	18	1.23	0.78–1.96	0.374	204	39	19
1–4.99	48	67	0.71	0.53–0.97	0.030	133	−54	−40
5+	115	115	1.00	0.82–1.23	0.985	209	0	0

Abbreviations: CI=confidence interval; HR=hazard ratio; PYAR=person-years at risk.

Reference is general population comparator cohort. The attributable percentage was calculated as (1–1/HR).

**Table 3:** Risk of prostate cancer (subgroup analyses)

**Subgroup**	**Observed events**	**Expected events**	**HR**	**HR; 95% CI**	***P*-value**	**Absolute risk/100 000 PYAR**	**Excess risk/100 000 PYAR**	**Attributable percentage**
*Age (years)*								
0–19	0	0	—	—				
20–39	3	1	3.05	0.74–12.56	0.123	18	12	67
40–59	71	69	1.03	0.79–1.33	0.841	285	7	3
60+	111	131	0.85	0.69–1.04	0.118	744	−133	−18
								
*Calendar period*								
−1989	49	50	0.97	0.71–1.34	0.873	164	−4	−3
1990–1999	95	103	0.92	0.74–1.15	0.464	181	−16	−9
2000–	41	46	0.89	0.64–1.24	0.504	211	−25	−12

Abbreviations: CI=confidence interval; HR=hazard ratio; PYAR=person-years at risk.

Reference is general population comparator cohort. The attributable percentage was calculated as (1–1/HR).
