# Supplementary material for: Risk of prostate cancer in a population-based cohort of men with coeliac disease
Source: Br J Cancer. 2011 Dec 1;106(1):217–21. doi: 10.1038/bjc.2011.536 (PMC3251872; doi:10.1038/bjc.2011.536)
Supplement: Supplementary Appendix [file bjc2011536x1.doc]

**ONLINE APPENDIX**

Log-minus-log curves show that the proportional hazards assumption is fulfilled.

**
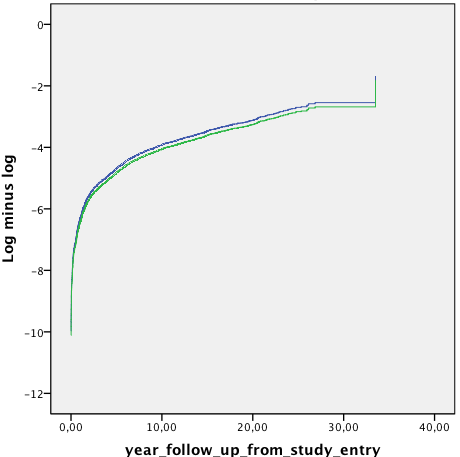
**

**Small intestinal histopathology classifications – a comparison**

| ***Classification used in this project*** | ***Villous atrophy*** | | |
| --- | --- | --- | --- |
| Marsh Classification* | Type 3a | Type 3b | Type 3c |
| Marsh  Description | Flat destructive | | |
| Corazza*et al* (*ref A*) | Grade B1 | | Grade B2 |
| SnoMed Codes | M58,  D6218,  M58005 | M58,  D6218,  M58006 | M58,  D6218,  M58007 |
| KVAST/Alexander classification | III  Partial VA | IV  Subtotal VA | IV  Total VA |
|  |  |  |  |
| *Characteristics* |  |  |  |
| Villous atrophy | + | ++ | ++ |
| IEL# | + | + | + |
| Crypt hyperplasia | + | ++ | ++ |

# Increased intraepithelial lymphocyte count (often >30/100 epithelial cells).

KVAST: Kvalitets- och Standardiseringskommittén (English: Committee for Quality and Standardization).

**ICD coding for type 1 diabetes mellitus**. Before 1997, the ICD coding for diabetes (ICD-7: 260, ICD-8: 250, ICD-9: 250) did not distinguish between type 1 and type 2 diabetes. We defined individuals with type 1 diabetes as those who were ≤ 30 years of age at their first hospitalization for diabetes (ICD-7-ICD-10).
